# Functional Neurologic Disorders: The Role of Limbic System

**DOI:** 10.1192/j.eurpsy.2024.727

**Published:** 2024-08-27

**Authors:** M. Lima Magalhães, M. Andrade, A. Lourenço, G. Soares

**Affiliations:** ^1^Clínica 6; ^2^Clínica 3, Centro Hospitalar Psiquiátrico de Lisboa, Lisboa, Portugal

## Abstract

**Introduction:**

Functional Neurological Disorders (FND), also called hysteria or conversion disorder, have represented a challenge over the centuries in terms of comprehension of the mechanisms responsible for symptoms which mimic neurological diseases without organic damage. Charcot considered hysteria primarily a hereditary disorder, but also considered that environmental factors including physical and emotional stress served as provoking factors. The prevailing etiologic theories of FND are psychosocial and still strongly dominated by the Freudian concept of conversion – a psychologic symptom is converted into a somatic symptom as a way of dealing with the distress of the symptom. However, physiologic studies with fMRI are necessary to understand the neurological mechanisms involved in FND symptoms. Convergent neuroimaging findings have implicated abnormal limbic-motor interactions in response to emotional stimuli in FND patients, demonstrated a possible role of the limbic system (LS) in FND neurophysiology.

**Objectives:**

Understand the role of LS in the neurophysiologic mechanisms involve in FND.

**Methods:**

Systematic review of the literature published in PubMed, using the terms **“Functional Neurological Disorders”**, **“Limbic System”**, **“Emotions”**.

**Results:**

Physiologic studies of functional weakness and sensory loss reveal normal functioning of primary motor and sensory cortex, but abnormalities of premotor cortex and association cortices. This suggests a top-down influence creating the dysfunction during the action control. Indeed, fMRI studies with FND motor patients show a hypoactivation of cortical and subcortical motor pathways, and a hyperactivity in limbic areas related with an abnormal limbic regulation with increased amygdala activity. In fact, studies have found a dysfunction in the medial prefrontal areas in FDN patients suggesting that they might have an abnormal affective representation (AR) of self-relevant information encoded in this region, which can later induce specific behavioral patterns of thought interaction with sensorimotor circuits. The abnormal AR could be influence by a dysfunction in LS regulation. Indeed, emotions are one of the major factors influencing movement choice. Moreover, limbic structures, such as the amygdala, can be influenced by genetic factors and/or early life stress. Thus, abnormal functioning of LS could lead to functional disorders by deranged top-down control.

**Conclusions:**

In conclusion, FND patients may have an abnormal AR and/or emotion regulation mechanisms possibly due to prior experience or partly genetically determined which interact with lower-order functions leading to the production of the functional symptoms, where LS have an important role. However, much further empiric research is needed to better understand this fascinating and debilitating condition, as well as to derive new perspectives for more efficient therapeutic interventions in these patients.

**Disclosure of Interest:**

None Declared

